# Band-on-band endoscopic variceal ligation: a technique for the treatment of esophageal varices in case of band misplacement

**DOI:** 10.1055/a-2268-5513

**Published:** 2024-03-01

**Authors:** Andrea Sorge, Tommaso Pessarelli, Luca Elli, Nicoletta Nandi, Giulia Tosetti, Massimo Primignani, Gian Eugenio Tontini

**Affiliations:** 1 Department of Pathophysiology and Transplantation, University of Milan, Milan, Italy; 2 9339 Gastroenterology and Endoscopy Unit, Fondazione IRCCS Caʼ Granda Ospedale Maggiore Policlinico, Milan, Italy; 3 Gastroenterology and Hepatology Unit, Fondazione IRCCS Caʼ Granda Ospedale Maggiore Policlinico,

A 62-year-old woman with porto-sinusoidal vascular disorder and portal hypertension was admitted to the emergency department for hematemesis and anemia (hemoglobin 8.1 mg/dL). Five years ago, she had been treated at another hospital with two sessions of endoscopic variceal ligation (EVL) for esophageal variceal bleeding. After hemodynamic stabilization, an upper gastrointestinal endoscopy was performed and large-caliber esophageal varices with red signs were found in the middle and lower third of the esophagus. There was no active bleeding. EVL (Speedband Superview Super 7; Boston Scientific, Marlborough, Massachusetts, United States) was performed 1 , but the placement of one of the bands failed due to poor tissue elevation, causing accidental oozing bleeding. Another band was promptly placed achieving hemostasis, but the position of the band was suboptimal due to inadequate tissue elevation. The small amount of variceal tissue grasped by the band could have caused premature band dislodgement and severe post-banding ulcer bleeding. Therefore, we placed the banding cap over the misplaced band ( Fig. 1 ) and applied prolonged suction to achieve maximal tissue prolapse inside the banding cap ( Video 1 ). Finally, we placed a new band below the previous one ( Fig. 2 ). Such band-on-band EVL was successful, achieving optimal band placement without residual bleeding. No further adverse events occurred and the patient was discharged home 4 days after admission. At a 1-month follow-up visit, no adverse events or rebleeding were reported.

**Fig. 1:**
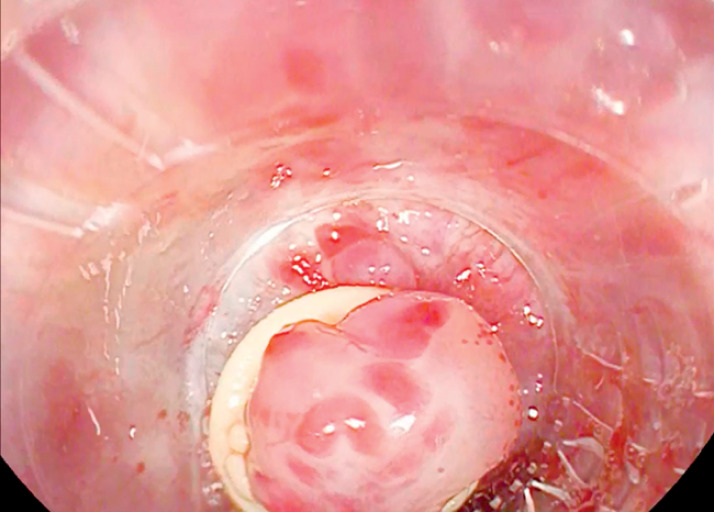
Banding cap applied over the misplaced band.

Band-on-band endoscopic variceal ligation in a patient with incomplete variceal suction and band misplacement.Video 1

**Fig. 2:**
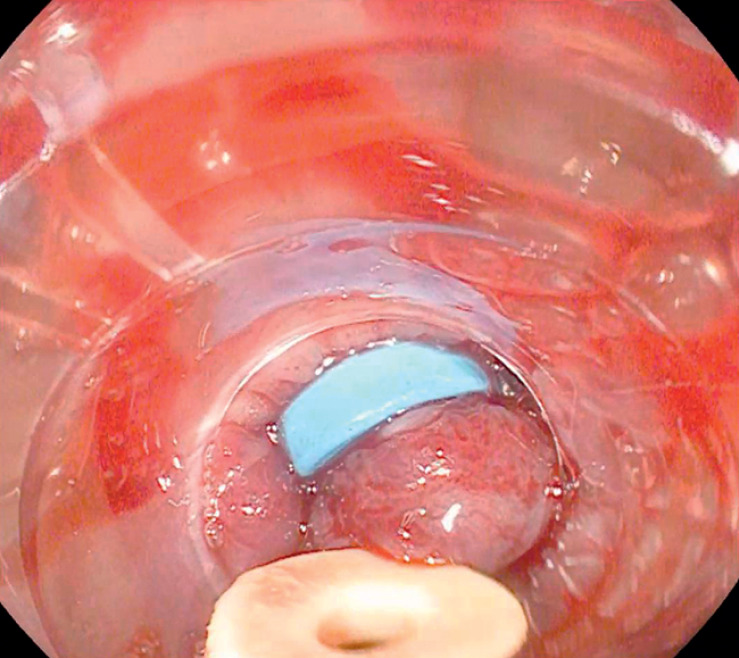
Optimal band placement after band-on-band ligation.

The success rate of EVL is about 85–94% 2 3 with incomplete suction due to fibrosis from previous endoscopic treatments being one of the major causes of failure 4 . This case describes for the first time the band-on-band EVL technique, a safe and effective method for the management of band misplacement during EVL. Band-on-band EVL could be a valid option to ensure optimal and durable treatment for incomplete variceal suction and band misplacement cases.

Endoscopy_UCTN_Code_TTT_1AO_2AD

## Notes

Endoscopy E-Videos https://eref.thieme.de/e-videos
*E-Videos* is an open access online section of the journal *Endoscopy* , reporting on interesting cases and new techniques in gastroenterological endoscopy. All papers include a high-quality video and are published with a Creative Commons CC-BY license. Endoscopy E-Videos qualify for HINARI discounts and waivers and eligibility is automatically checked during the submission process. We grant 100% waivers to articles whose corresponding authors are based in Group A countries and 50% waivers to those who are based in Group B countries as classified by Research4Life (see: https://www.research4life.org/access/eligibility/ ). This section has its own submission website at https://mc.manuscriptcentral.com/e-videos .
